# Cardiac and electro-cortical concomitants of social feedback processing in women

**DOI:** 10.1093/scan/nsv039

**Published:** 2015-04-13

**Authors:** Laura M. S. Dekkers, Melle J. W. van der Molen, Bregtje Gunther Moor, Frederik M. van der Veen, Maurits W. van der Molen

**Affiliations:** ^1^Department of Psychology, University of Amsterdam, Weesperplein 4, 1018 XA Amsterdam, The Netherlands,; ^2^Department of Psychology, Leiden University, Wassenaarseweg 52, 2333 AK Leiden, The Netherlands,; ^3^Leiden Institute for Brain and Cognition, Leiden University Medical Center, Postzone C2-S, P.O. Box 9600, 2300 RC Leiden, The Netherlands,; ^4^Institute of Psychology, Erasmus University Rotterdam, Woudsestein T13-1, 3000 DR Rotterdam, The Netherlands, and; ^5^Department of Psychiatry, Erasmus Medical Centre, P.O. Box 2040, 3000 CA Rotterdam, The Netherlands

**Keywords:** social feedback processing, transient cardiac slowing, event-related potentials, P3, feedback-related negativity

## Abstract

This study provides a joint analysis of the cardiac and electro-cortical—early and late P3 and feedback-related negativity (FRN)—responses to social acceptance and rejection feedback. Twenty-five female participants performed on a social- and age-judgment control task, in which they received feedback with respect to their liking and age judgments, respectively. Consistent with previous reports, results revealed transient cardiac slowing to be selectively prolonged to unexpected social rejection feedback. Late P3 amplitude was more pronounced to unexpected relative to expected feedback. Both early and late P3 amplitudes were shown to be context dependent, in that they were more pronounced to social as compared with non-social feedback. FRN amplitudes were more pronounced to unexpected relative to expected feedback, irrespective of context and feedback valence. This pattern of findings indicates that social acceptance and rejection feedback have widespread effects on bodily state and brain function, which are modulated by prior expectancies.

## INTRODUCTION

People are strongly motivated to gain social acceptance and are typically highly sensitive to interpersonal rejection. Indeed, social rejection is conceptualized as a significant threat to survival (Baumeister and Leary, 1995; MacDonald and Leary, 2005). To explore its underlying neural correlates, Eisenberger *et al.* (2003) conducted a study in which participants were playing ‘Cyberball’—a virtual ball-tossing game in which participants get ostracized—in the scanner. Results revealed that the dorsal anterior cingulate cortex (dACC) and anterior insula—regions involved in physical pain processing (Shackman *et al.*, 2011)—and the right ventral prefrontal cortex—a region involved in controlling negative emotions (Agustín-Pavón *et al.*, 2012)—were more active during exclusion relative to inclusion episodes. Since this seminal study, others replicated and extended this pattern of findings (Eisenberger, 2012; Gunther Moor *et al.*, 2012; Cristofori *et al.*, 2013).

The importance of prior expectancies in modulating the brain’s response to social exclusion has been reinforced by studies using a paradigm that more explicitly manipulates social acceptance and rejection—the social-judgment paradigm. That is, Somerville *et al.* (2006) asked participants to decide whether they expected to be liked or disliked by peers that were presented to them on photographs. After each judgment, participants were provided with fictitious feedback signalling social acceptance or rejection. This design allowed for the examination of neural activity associated with social evaluative feedback (i.e. acceptance *vs* rejection) and expectancy violation (i.e. expected *vs* unexpected). Results revealed that processing of social acceptance feedback, relative to social rejection feedback, was accompanied by increased ventral ACC (vACC) activity. Processing of social feedback that violated prior expectancies, regardless of whether it signalled acceptance or rejection, was accompanied by increased dACC activity. This double dissociation led authors to argue that dACC activity to social exclusion reported in Cyberball studies is likely to reflect a violation of the fundamental expectancy of social inclusion (Somerville *et al.*, 2006; also see Gunther Moor *et al.*, 2010b).

Employing an extended version of the social-judgment paradigm, Gunther Moor *et al.* (2010a; 2014) examined cardiac responses to social evaluative feedback. They additionally used a non-social control task in which participants were asked to decide whether persons on the photographs were more than 21 years of age or not. Consistent with cardiac studies on processing of performance feedback (Crone *et al.*, 2003; Groen *et al.*, 2007; Luman *et al.*, 2007, 2008), results showed anticipatory heart rate deceleration to all feedback conditions in both tasks. Cardiac slowing was continued, however, when the feedback communicated unexpected social rejection (also see, van der Veen *et al.*, 2014). This prolonged cardiac slowing to unexpected social rejection feedback was interpreted as a cardiovagal manifestation of the central-autonomic network (Berntson *et al.*, 1993) implicated in the processing of relevant social information (Gunther Moor *et al.*, 2010a).

Prolonged cardiac slowing to unexpected social rejection feedback was replicated by van der Veen *et al.* (2014) who, additionally, examined electro-cortical responses to social evaluative feedback. They observed that a P3-like positive deflection—peaking ∼325 ms post-feedback at fronto-central electrode sites—was most pronounced to expected social acceptance feedback. Using a similar paradigm, Sun and Yu (2014) also observed more positive amplitudes to social acceptance feedback during a 300–400 ms post-feedback window. Consistent with suggestions that the P3 is sensitive to the motivational significance of a stimulus (Nieuwenhuis *et al.*, 2005; Polich, 2007), van der Veen *et al.* (2014) interpreted the enhanced P3-like response to expected social acceptance feedback in terms of an electro-cortical manifestation of a social bias; that is, confirmation of individuals’ typical expectation to be liked is socially rewarding.

Extending these initial electro-cortical findings, van der Molen *et al.* (2014) additionally examined the feedback-related negativity (FRN) to social evaluative feedback. The FRN is a negative deflection of the brain potential—maximal at ∼250 ms after feedback onset (Miltner *et al.*, 1997)—that has been extensively studied in performance monitoring paradigms (for a review see, Ullsperger *et al.*, 2014). Van der Molen *et al.* (2014) failed to observe the enhanced P3 to social acceptance feedback reported by van der Veen *et al.* (2014). However, they did observe an interesting, albeit marginally significant, FRN pattern. That is, FRN amplitudes tended to be larger to unexpected as compared with expected social evaluative feedback, irrespective of its valence. Kujawa *et al.* (2014) also examined the FRN to social evaluative feedback using a task in which participants could be voted out of a game by their peers. These authors observed larger FRN amplitudes to social rejection as compared with social acceptance feedback. However, in this study, FRN sensitivity to feedback valence (i.e. acceptance *vs* rejection) may have been confounded by expectancy, as expected and unexpected feedback could not be dissociated. Moreover, neither of the electro-cortical studies carried out thus far allows for conclusions on the social impact of the feedback *per se*, as effects of social evaluative feedback were not compared with effects of non-social feedback.

Employing both a social- and non-social age-judgment task (Gunther Moor *et al.* 2010a, 2014), this study aimed at providing a detailed analysis of the cardiac, P3 and FRN responses to social *vs* non-social feedback. We tested three hypotheses. First, we anticipated to replicate the previously found prolonged cardiac slowing to unexpected social rejection feedback relative to other types of social feedback (Gunther Moor *et al.*, 2010a; 2014; van der Veen *et al.*, 2014). Second, we anticipated to observe an enhanced P3 to expected social acceptance feedback as compared with other types of social feedback (van der Veen *et al.*, 2014; but see, van der Molen *et al.*, 2014) and tested whether this P3 response to social feedback is larger when compared with (expected) non-social feedback, thereby assuming that social feedback is more salient than non-social feedback (Somerville *et al.*, 2010). Third, we contrasted three competing hypotheses concerning FRN sensitivity to social feedback and tested whether the FRN response to social feedback differs from the FRN response to non-social feedback. Specifically, as suggested by the findings of van der Molen *et al.* (2014; also see, Hajcak *et al.*, 2007; Oliveira *et al.*, 2007; Ferdinand *et al.*, 2012), we could expect the FRN to be sensitive to feedback congruence, with an enhanced FRN to feedback that violates prior expectancies, irrespective of its valence. Alternatively, as suggested by the findings of Kujawa *et al.* (2014), we could expect the FRN to be sensitive to feedback valence, with an enhanced FRN to social rejection feedback. Finally, based on literature indicating that ACC regions are involved in the generation of the FRN (van Veen and Carter, 2002; Ridderinkhof *et al.*, 2004) and parasympathetic cardiac control (Porges, 2001; Critchley *et al.*, 2003; Lane *et al.*, 2004; Matthews *et al.*, 2004), we could expect that FRN responds to social feedback in the same way as heart rate. In this case, we would observe an interaction between feedback congruence and valence, with the most pronounced FRN to unexpected social rejection feedback. If supported, existing models of the FRN should be revised, reconciling congruence and valence interpretations, to allow for a ‘third way’ if feedback is of social evaluative nature.

## MATERIALS AND METHODS

### Participants

Twenty-five female university students (age: 18–27; *M* = 21.59; s.d. = 2.19) participated in the study.^1^ All participants had normal or corrected-to-normal vision. Exclusion criteria were self-reported current neurological or psychiatric illness, use of illicit drugs on a regular basis and use of prescribed medication. One participant was excluded from all analyses, because of a later reported current psychiatric illness. Three participants were excluded from the electroencephalography (EEG) data analysis due to uncorrectable artifacts in their EEG time series. Participants received a fixed payment or course credits. Prior to participation, written consent was obtained. The study was approved by the local Ethics Committee of the University.

### Stimulus materials, task description and experimental design

Participants were informed that they were enrolled in a study on first impressions. They were asked to send their portrait photograph to one of the researchers. Participants were told that peers at other universities would form impressions about their photographs before visiting the lab. Photographs of these peers would be presented to them during the test session when they would have to perform two tasks in which they had to form impressions about these peers. Unbeknownst to the participants, peers did not judge their portrait photographs and the photos presented to them during the test session were of volunteers who provided written consent to use of their photograph for scientific purposes.

During the test session, participants observed neutral faces of age-matched peers (age: 17–29; Male: *M* = 22.64, s.d. = 2.45; Female: *M* = 20.90, s.d. = 2.08). Faces (width = 0.039° VA, height = 0.055° VA) were presented using Presentation software (version 14.2; Neurobehavioral Systems, Albany, CA) in color against a black background in the center of a 22-inch computer monitor (refresh rate = 60 Hz, resolution = 1600 × 900 pixels). Both tasks consisted of the same photographs of 170 different faces with an equal distribution of male and female faces.

**Fig. 1:**
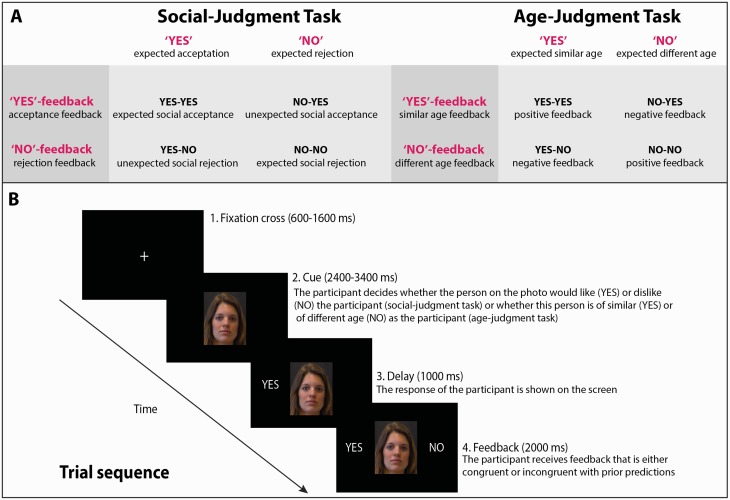
Schematic of experimental design and trial sequence. A: Schematic of experimental design. Note that tasks differed in what constituted negative feedback. In the social-judgment task, participants received negative feedback in the ‘Yes’–‘No’ and ‘No’–‘No’ conditions, with ‘No’ communicating social rejection; they received positive feedback in the ‘Yes’–‘Yes’ and ‘No’–‘Yes’ conditions, with ‘Yes’ communicating social acceptance. In the age-judgment task, however, participants received negative feedback in the ‘Yes’–‘No’ and ‘No’–‘Yes’ conditions, where they incorrectly judged the peer presented to them to be of the same or a different age, respectively; they received positive feedback in the ‘Yes’–‘Yes’ and ‘No’–‘No’ conditions, where they correctly judged the peer to be of the same or a different age, respectively. B: Example of a trial sequence (‘Yes’–‘No’ condition) in the social- and age-judgment task. Trials always started with a centrally depicted fixation cross, with a 600–1600 ms jittered duration. The fixation cross was followed by a facial stimulus that remained on the screen for the rest of the trial. During a jittered response window of 2400–3400 ms, participants were asked to respond ‘Yes’ or ‘No’ to communicate their judgment. Duration of the fixation cross and response window was linked, so that summing the presentation times of both always added up to 4000 ms. Responses (‘Yes’/‘No’) appeared on the screen, after the response window was terminated, at the left side of the face, during a period of 1000 ms. Subsequently, feedback (‘Yes’/‘No’) appeared on the screen, at the right side of the face, during a period of 2000 ms. When responses were not made within the response window, the feedback ‘Too Slow’ appeared, which was followed by the initiation of a new trial. Total trial duration was fixed at 6000 ms.

Figure 1A depicts a schematic of the experimental design. Participants performed on two tasks. In the social-judgment task, participants were asked to decide whether they expected to be liked (‘Yes’-response) or not (‘No’-response); in the age-judgment task they were asked to indicate whether they thought that the person on the picture is of their age (‘Yes’-response) or not (‘No’-response). After each judgment, participants were provided feedback signalling social acceptance (‘Yes’-feedback) or rejection (‘No’-feedback) in the social-judgment task or indicating whether the peer indeed was of their age (‘Yes’-feedback) or a different age (‘No’-feedback) in the age-judgment task. An example of a trial sequence is presented in Figure 1B.

Participants were asked to communicate their judgment by pressing a button on the left or right armchair. The order of both tasks and responding hand for ‘Yes’- and ‘No’-responses were counterbalanced across participants. In both tasks, participants performed on 10 practice trials and four successive blocks containing 40 test trials. On half of the trials they received ‘Yes’-feedback; on the other half of the trials they received ‘No’-feedback. Unbeknownst to the participants, feedback for all trials was generated pseudo-randomly by the computer.

### Procedure

Electrocardiography (ECG) and EEG equipment was attached after participants signed the informed consent form and were reminded to the purpose of the study by a rehearsal of the cover story. Participants were tested in a sound and electrical shielded EEG chamber, while sitting at a distance of ∼75 cm from the computer monitor. Prior to each task, participants received verbal and written instructions on the task at hand and baseline electrophysiological measures were recorded during a 2-min period. Between tasks, participants were permitted a 5-min rest. After the ECG/EEG session, IQ was assessed and the self-report questionnaires were administered (for details, see Supplementary Material). Thereafter participants were asked to write down their experiences during and thoughts about the study, to test for the validity of the cover story. On the basis of the information thus collected, participants seemed unaware of the purpose of the study; none of them reported any doubts about the cover story. Participants received a debriefing letter after all participants had been tested.

### Data recording

ECG and EEG were recorded continuously at a sampling frequency of 1024 Hz with a 64-channel ActiveTwo system (BioSemi, Amsterdam, The Netherlands), using Ag-AgCl (silver-silver chloride) electrodes. ECG electrodes were placed at the sternum and the rib above the lowest rib at the left side of the body; 64 EEG electrodes were mounted in an elastic electrode cap (10/20 system). Electrode offsets were, on average, kept below 30 µV. The BioSemi common mode sense (CMS) active electrode and driven right leg (DRL) passive electrode were used as grounds; CMS was used as online reference. Horizontal and vertical electro-oculography (EOG) was measured with two Ag-AgCl electrodes placed on the left and right cantus and above and below the left eye, respectively.

### Data reduction

#### Electrocardiography

The ECG signal was offline filtered with a high-pass filter of 20 Hz and exported to PhysioSpec (in house software) for extracting interbeat intervals (IBIs). IBIs reflect the time interval in millisecond between two individual heart beats and constitute a chronotropic measure of heart rate that has been frequently used to assess stimulus anticipation and processing (for a review see, Jennings and van der Molen, 2005). R-peaks were identified in case a peak in the ECG signal occurred in the highest 25% of the range of the signal, with the restriction that the time interval between two consecutive R-peaks could neither be smaller than 400 ms or larger than 1400 ms. All selected R-peaks were manually screened and corrected if necessary. Subsequently, the IBI concurrent with the feedback (IBI 0), 2 pre-feedback IBIs (IBI-2, IBI-1) and 4 post-feedback IBIs (IBI 1 to IBI 4) were selected (Gunther Moor *et al.*, 2010a). All selected IBIs were referenced to the second pre-feedback IBI pre (i.e. IBI-2). Preliminary analyses on pre-feedback IBIs did not result in any differences across tasks or conditions, *P*s > 0.05.

#### Electroencephalography.

Offline analysis of the EEG time series was performed using Brain Vision Analyzer (version 1.05.0005, Brain Products GmbH, 1998–2007). EEG time series were downsampled to 512 Hz, rereferenced to the left and right mastoids and filtered with a 0.1–30 Hz (24 dB/oct) band-pass and 50 Hz notch filter. EOG artifacts were removed from the data using the Gratton *et al.* (1983) regression procedure. Bad channels were interpolated with neighboring channels. Subsequently, 7000 ms epochs were created time-locked to the onset of the facial stimulus, including a 500 ms pre-stimulus interval. Since gross disturbances at the time of stimulus presentation and response as well as feedback presentation could have influenced feedback processing, epochs, encompassing the entire trial, were used to inspect the EEG time series for artifacts. Epochs with a signal exceeding a maximal voltage step of 50 µV and/or in which the lowest allowed activity in a time window of 100 ms did not exceed 0.50 µV were rejected automatically. Epochs were thereafter visually inspected for additional artifacts. The number of kept trials ranged from 10 to 55 (*M* = 35.30, s.d. = 1.40) and 19 to 55 (*M* = 36.49, s.d. = 0.79) for conditions in the social- and age-judgment task, respectively. Artifact-free epochs were segmented in 1000 ms epochs time-locked to the feedback onset, including a 200 ms pre-feedback period that was used for baseline correction. Preliminary analyses on baseline activity did not result in any differences across tasks or conditions, *P*s > 0.05.

The grand averages prompted us to look at two P3 measures, an early and a late P3. As P3 tends to be most pronounced at centroparietal electrode positions (Polich, 2007), both were determined at C3, Cz, C4, P3, Pz, P4, O1, Oz and O2. Early P3 peak amplitudes were defined base-to-peak as the most positive value of the event-related potential (ERP) within the 280–500 ms post-feedback window relative to the 200 ms pre-feedback baseline corrected base. The latency at which the P3 reached peak amplitude was taken as P3 peak latency. Late P3 was defined by mean voltage within the 425–650 ms post-feedback window.

As the FRN typically reaches maximum amplitudes at frontocentral electrode positions (Ullsperger *et al.*, 2014), FRN peak amplitude was computed at F3, Fz, F4, FC3, FCz, FC4, C3, Cz and C4 by (i) identifying P2 amplitude (i.e. the most positive value in the 150–250 ms post-feedback window) as the onset of the negativity, (ii) determining the most negative value within a window determined from the onset of the negativity until 350 ms post-feedback (i.e. FRN time-window) and (iii) taking the difference between these values as FRN amplitude (Holroyd *et al.*, 2003). The FRN was scored 0 µV when no negativity could be identified within the FRN time window. The latency at which peak negativity was found was taken as FRN peak latency.

### Statistical analyses

We adopted a two-step procedure (cf., Gunther Moor *et al.*, 2010a; 2014). That is, for each electrophysiological measure (i.e. dependent variables of interest; IBI, FRN amplitude, FRN latency, early P3 amplitude, early P3 latency, late P3 amplitude), we first tested its pattern to different types of social feedback, by performing a repeated measures analysis of variance (ANOVA), with Congruency (2 levels; incongruent, congruent) and Feedback Type (2 levels; Yes, No) as within-subjects factors, for the social-judgment task. For the cardiac analysis, sequential IBI (3 levels, IBI 1, IBI 2, IBI 3) was included as a third within-subjects factor. We then performed a similar ANOVA for the age-judgment task. In the case these separate analyses revealed consistent effects for both tasks, we performed a repeated measures ANOVA, with Task (2 levels; social, age), Congruency (2 levels; incongruent, congruent) and Feedback Type (2 levels; Yes, No) as within-subjects factors, to test whether these effects were modulated by context (i.e. Task). This two-step analytical strategy was adopted as conditions differed across the social- and age-judgment task (Figure 1A). In addition, valence (acceptance-positive *vs* rejection-negative) and congruency (expected *vs* unexpected) could only be dissociated in the social- but not age-judgment task.

Statistical analyses were carried out with IBM SPSS Statistics 20 (IBM Corporation, 1989–2011). Results were evaluated against an alpha of 0.05. To preserve power in case of violation of sphericity, results of multivariate tests were evaluated.

## RESULTS

### Behavior

We examined potential response bias by examining the frequency of ‘Yes’- *vs* ‘No’-judgments for each task. As can be seen in Table 1, this analysis indicated that, in the social-judgment task, participants more often predicted to be liked (i.e. ‘Yes’-judgments) than disliked (i.e. ‘No’-judgments), *t*(23) = 2.48, *P* = 0.02, *d* = 1.01. Response bias was absent in the in the age-judgment task, *P* > 0.05. For results on response latency, see supplementary material.

**Table 1:** Mean (s.d.) number of trials and response latencies within each condition in the social- and age-judgment task.

	Social-judgment task		Age-judgment task	
	Frequency	Response time	Frequency	Response time
Yes	86.29 (14.44)	1296.04 (274.39)	73.92 (14.21)	1274.63 (237.63)
Yes–Yes	43.13 (8.35)		36.29 (8.55)	
Yes–No	43.17 (7.71)		37.62 (6.51)	
No	71.96 (14.00)	1302.38 (241.94)	84.79 (14.49)	1245.92 (227.86)
No–No	35.83 (7.60)		41.83 (6.79)	
No–Yes	36.13 (7.93)		42.96 (8.67)	

Note that mean (s.d.) number of trials and response latencies are based on data of 24 participants, included in het cardiac analyses. For the electro-cortical analyses, these numbers were slightly, but non-significantly different, due to exclusion of three participants (i.e. *N* = 21) and rejection of bad trials during preprocessing.

### Electrocardiography

Preliminary analyses on trial numbers within conditions of both tasks revealed that, although the interaction between Tasks and Condition reached significance, *F*(3, 21) = 4.62, *P* = 0.012, ${\text{η}}_{p}^{2}$> = 0.40, there were no significant differences between conditions as indicated by *post hoc* tests separated by Task, *P*s > 0.05.

**Fig. 2:**
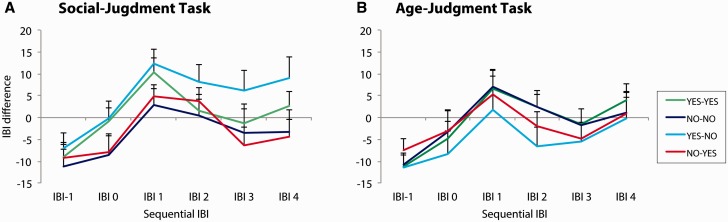
IBI difference scores. A: IBI difference scores for sequential IBIs for each condition in the social-judgment task. B: IBI difference scores for sequential IBIs for each condition in the age-judgment task. Error bars indicate SEM. The pattern reveals that, for both tasks, IBIs lengthen (i.e. heart rate slows) in anticipation of the feedback stimulus and returns to baseline following its onset. The return to baseline seems delayed for unexpected social rejection feedback (‘Yes’–‘No’ condition) in the social-judgment task (A) (see main text).

**Fig. 3:**
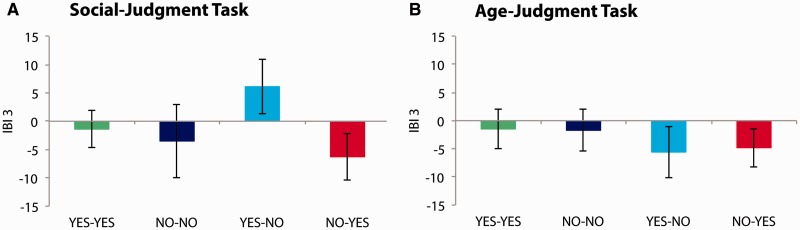
IBI difference scores for the third IBI contingent upon the feedback stimulus. A: IBI difference scores for the third IBI contingent upon the feedback stimulus (i.e. IBI 3) for each condition in the social-judgment task. B: IBI difference score for the third IBI contingent upon the feedback stimulus (i.e. IBI 3) for each condition in the age-judgment task. Error bars indicate SEM. As illustrated in this Figure, the feedback effect on IBI 3 has a negative value in all conditions, with the exception of the ‘Yes’–‘No’ condition in the social-judgment task (A). For unexpected social rejection feedback, IBI 3 has a positive value, indicating a transient delay in the recovery to baseline heart rate.

The cardiac response to feedback is presented in Figure 2. For the social-judgment task, the repeated measures ANOVA, yielded a Congruency by Feedback Type interaction, *F*(1, 23) = 7.22, *P* = 0.01, ${\text{η}}_{p}^{2}$> = 0.24, that was included in a Congruency by Feedback Type by IBI interaction, *F*(2, 22) = 5.18, *P* = 0.01, ${\text{η}}_{p}^{2}$> = 0.32; other *P*s > 0.05. Subsequent analyses on separate IBIs indicated that IBI 3 associated with unexpected social rejection feedback (‘Yes’–‘No’) was longer than the corresponding IBI associated with unexpected social acceptance feedback (‘No’–‘Yes’), *t*(23) =−3.16, *P* = 0.004, *d* = 0.56. None of the other pairwise comparisons reached significance, *P*s > 0.05. The effect of feedback on IBI 3 is presented in Figure 3 for illustrative purposes only. A similar analysis for the age-judgment task revealed that the IBI response did not discriminate between conditions in this task, *P* > 0.05.

### Electroencephalography

**Fig. 4:**
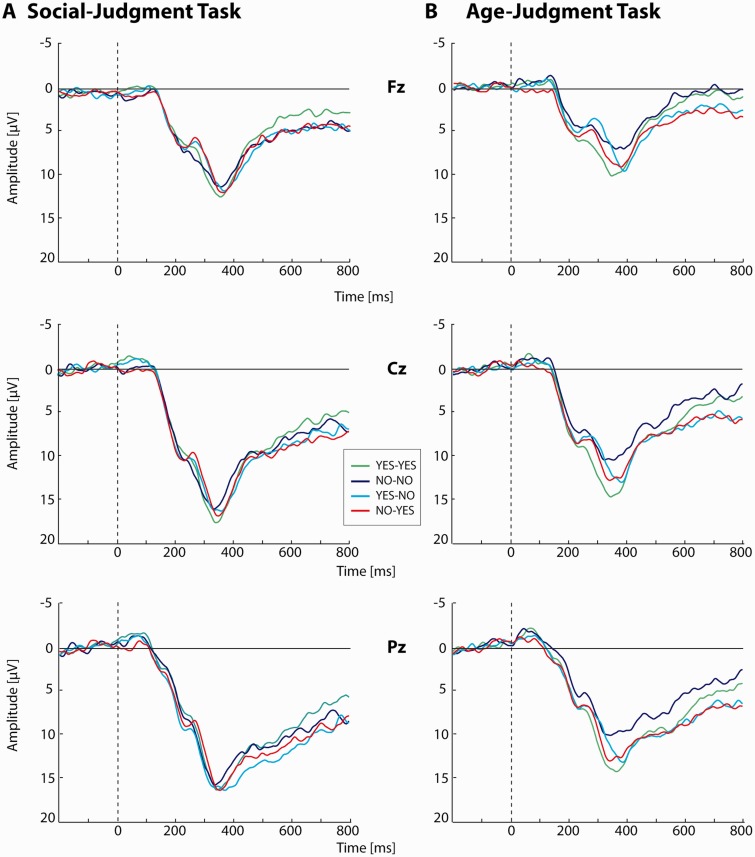
Grand average ERP waveforms. A: Grand average ERP waveforms for the social-judgment task at Fz (upper panel), Cz (middle panel) and Pz (lower panel). B: Grand average ERP waveforms for the age-judgment task at Fz (upper panel), Cz (middle panel) and Pz (lower panel). As the FRN and early and late P3 were analyzed at Fz and Pz, respectively, were they reached maximal amplitudes in all conditions and both tasks, ERP waveforms at Cz are displayed for illustrative purposes only.

The grand average ERP waveforms associated with the feedback stimulus at Fz, Cz and Pz are presented in Figure 4. Preliminary analyses on trial numbers within conditions of both tasks revealed that, although the interaction between Task and Condition reached significance, *F*(3, 18) = 3.67, *P* = 0.03, ${\text{η}}_{p}^{2}$> = 0.39, there were no significant differences between conditions, as indicated by *post hoc* tests separated by Task, *P*s > 0.05.

We report results for early and late P3 amplitudes at Pz and FRN amplitudes at Fz, as amplitudes were maximal at these sites for all conditions and both tasks.^2^ For voltage and current source density scalp maps of P3 and FRN activity as well as results on P3 and FRN latencies, see supplementary material.

#### P3 amplitude

**Fig. 5:**
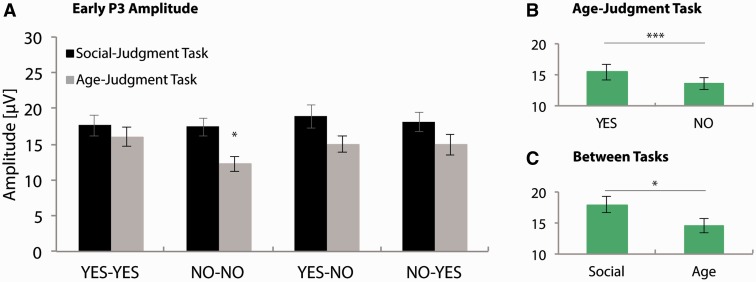
Early P3 peak amplitudes at Pz. A: Average early P3 peak amplitudes at Pz, in the 280–500 ms post-feedback window, for all conditions in the social- and age-judgment task. * = 0.05 > *P* > 0.005; Early P3 peak amplitude to the ‘No’-‘No’ condition of the age-judgment task was significantly smaller than early P3 peak amplitudes to all other conditions in this task (for details, see main text). B–C: Significant main effects (for details, see main text). * = 0.05 > *P* > 0.005; ** = 0.005 > *P* > 0.001; *** = *P* < 0.001. Error bars indicate SEM.

**Fig. 6:**
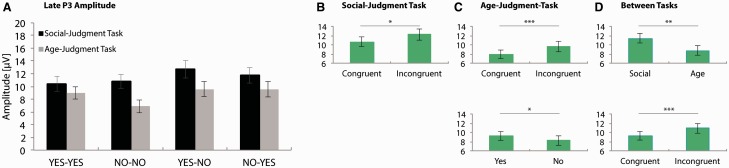
Late P3 mean amplitudes at Pz. A: Average late P3 activity at Pz, capturing voltage in the 425–650 ms post-feedback window, for all conditions in the social- and age-judgment task. B–D: Significant main effects (for details, see main text). * = 0.05 > *P* > 0.005; ** = 0.005 > *P* > 0.001; *** = *P* < 0.001. Error bars indicate SEM.

Early and late P3 amplitudes at Pz are presented in Figures 5 and 6, respectively. For the social-judgment task, the ANOVA on early P3 peak amplitudes failed to reveal significant differences between conditions, *P*s > 0.05. For the age-judgment task, a main-effect of Feedback Type was found, *F*(1, 20) = 7.13, *P* = 0.02, ${\text{η}}_{p}^{2}$> = 0.26, that was included in a Congruency by Feedback Type interaction, *F*(1, 20) = 6.04, *P* = 0.02, ${\text{η}}_{p}^{2}$> = 0.23. Paired-samples *t*-tests revealed that the P3 peak amplitude in the ‘No’-‘No’ condition was smaller than the P3 peak amplitude in all other conditions of this task, ‘Yes’–‘Yes’, *t*(20) = −3.20, *P* = 0.01, *d* = 0.69; ‘Yes’–‘No’, *t*(20) = −2.96, *P* = 0.01, *d* = 0.54; ‘No’–‘Yes’, *t*(20) = −3.13, *P* = 0.01, *d* = 0.47. Finally, we carried out a *post hoc* analysis across tasks to verify whether the context dependence that was observed for the late P3 (see later) was also present for the early P3. This analysis revealed that early P3 amplitudes were more pronounced to social (17.96 [1.29]) when compared with non-social feedback (14.53 [1.11]), *F*(1, 20) = 15.84, *P* = 0.001, ${\text{η}}_{p}^{2}$> = 0.44.^3^

Similar analyses were performed on late P3 amplitudes. For the social-judgment task a main-effect of Congruency was found, *F*(1, 20) = 5.82, *P* = 0.03, ${\text{η}}_{p}^{2}$> = 0.23. Late P3 amplitude was larger to unexpected (12.21 [1.21]) relative to expected feedback (10.61 [1.06]), other *P*s > 0.05. For the age-judgment task the analysis showed main effect of both Congruency, *F*(1, 20) = 15.94, *P* = 0.001, ${\text{η}}_{p}^{2}$> = 0.44, and Feedback Type, *F*(1, 20) = 5.01, *P* = 0.04, ${\text{η}}_{p}^{2}$> = 0.20. Late P3 was larger to unexpected (9.56 [1.13]) when compared with expected feedback (7.94 [0.91]) and to feedback indicating that the person on the photograph was of the same age as the participant (‘Yes’; 9.27 [1.02]) relative to feedback indicating that the person on the photograph was of a different age as the participant (‘No’; 8.23 [1.04]).

Finally, the subsequent test across tasks yielded main-effects of Task, *F*(1, 20) = 17.59, *P* < 0.001, ${\text{η}}_{p}^{2}$> = 0.47 and Congruency, *F*(1, 20) = 12.91, *P* = 0.002, ${\text{η}}_{p}^{2}$> = 0.39; other *P*s > 0.05. Late P3 was larger to social (11.41 [1.09]) when compared with non-social feedback (8.75 [1.01]) and following unexpected (10.89 [1.11]) relative to expected feedback (9.28 [0.93]), though this later effect was not modulated by context.

#### FRN amplitude

**Fig. 7:**
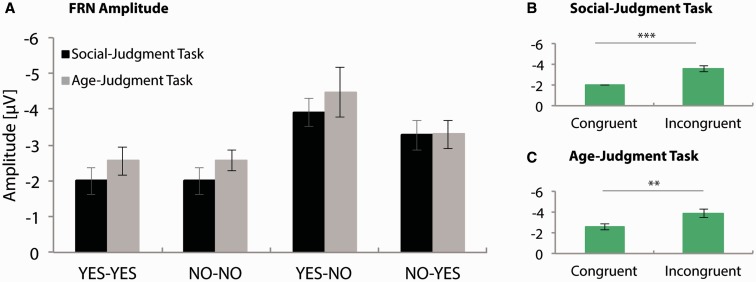
FRN peak amplitudes at Fz. A: Average FRN amplitudes at Fz for all conditions in the social- and age-judgment task. B–C: Significant main effects (for details, see main text). * = 0.05 > *P* > 0.005; ** = 0.005 > *P* > 0.001; *** = *P* < 0.001. Error bars indicate SEM. Note that FRN amplitude was defined as a relative measure, creating the possibility that the condition with the absolute most negative deflection in the ERP (Figure 4) is not necessarily the condition in which the most pronounced FRN amplitude was observed.

FRN amplitudes at Fz are presented in Figure 7. ANOVAs for both the social- and age-judgment task, only yielded a main-effect of Congruency, *F*(1, 20) = 17.44, *P* < 0.001, ${\text{η}}_{p}^{2}$> = 0.47; *F*(1, 20) = 9.68, *P* = 0.005, ${\text{η}}_{p}^{2}$> = 0.33, respectively; other *P*s > 0.05. For both tasks, FRN amplitudes were more pronounced to unexpected (social, −3.58 [0.35]; age, −3.89 [0.41]) relative to expected feedback (social, −1.99 [0.31]; age, −2.56 [0.28]). Subsequent tests revealed that the effect of Congruency did not differ between tasks, *F*(1, 20) = 23.58, *P* < 0.001, ${\text{η}}_{p}^{2}$> = 0.54; other *P*s > 0.05.

### Correlational analyses

Correlational analyses failed to show any systematic relationship between cardiac and electro-cortical responses to either social or non-social feedback (for details, see supplementary material).

## DISCUSSION

This study examined the cardiac and electrocortical concomitants of social feedback processing. Consistent with previous work (Crone *et al.*, 2003; Groen *et al.*, 2007; Luman *et al.*, 2007, 2008), we observed heart rate slowing in anticipation to feedback that returned to baseline following its onset. The acceleratory recovery toward baseline was delayed to feedback communicating unexpected social rejection. This prolonged cardiac slowing is consistent with previous studies (Gunther Moor *et al.*, 2010a, 2014; van der Veen *et al.*, 2014; Papousek *et al.*, 2014) and has been interpreted in terms of a cardiovagal response to relevant social cues (Gunther Moor *et al.*, 2010a).

Our P3 findings differ from those reported by van der Veen *et al.* (2014; but see, van der Molen *et al.*, 2014) in that we did not observe an enhanced P3 to expected social acceptance feedback. We did observe, however, that both early and late P3 were enhanced to social relative to non-social feedback and that late P3, in both tasks, was larger to unexpected when compared with expected feedback. This pattern extends the findings of van der Veen *et al.* (2014) and provides support for their saliency account. That is, the contextual influence on both early and late P3 is consistent with reports of larger P3 and late positive potential amplitudes to emotional (for a review, see Hajcak *et al.*, 2010) and self-relevant stimuli (Gray *et al.*, 2004). In this regard, the current findings suggest that social cues may capture attention and, thus, attract more processing resources (cf., Wu and Zhou, 2009; Gu *et al.*, 2011) resulting in amplified electrocortical responses (for a review see, Hajcak *et al.*, 2010).

Our FRN findings are consistent with those of van der Molen *et al.* (2014) who observed that the FRN tended to be larger to unexpected relative to expected feedback, irrespective of its valence. Our results revealed a statistically robust pattern and, additionally indicated that the FRN is not sensitive to context, in that the FRN pattern did not discriminate between the social *vs* non-social feedback. It could be argued that the current findings diverge from those reported by Kujawa *et al.* (2014) who observed a larger FRN to social feedback of negative relative to positive valence. This is not necessarily the case, however. Given that participants tend to expect they are liked (or will be voted in) (Gunther Moor *et al.*, 2010a; 2014; also see current data) social rejection feedback, indicating to be voted out from the game, violates expectancy. Thus, it is difficult to disentangle the contribution of congruency *vs* valence to the FRN findings presented by Kujawa *et al.* (2014) (see Somerville *et al.*, 2006 for a similar argument when discussing neuroimaging findings from Cyberball studies). Finally, ACC regions are involved in the generation of FRN (van Veen and Carter, 2002; Ridderinkhof *et al.*, 2004) and parasympathetic cardiac control (Porges, 2001; Critchley *et al.*, 2003; Lane *et al.*, 2004; Matthews *et al.*, 2004). However, the differential sensitivity of these measures to social evaluative feedback we observed, together with the absence of correlations amongst them (also see, van der Veen *et al.*, 2004), suggest that they are manifestations of distinct underlying processes.

Collectively, the current FRN findings speak to the congruency (Alexander and Brown, 2010) *vs* valence (Yeung and Sanfey, 2004) account known from the performance monitoring literature. Previously, it has been argued that FRN sensitivity to feedback valence could be due to event probability, with negative feedback typically having a lower probability of occurrence than positive feedback (for a review see, San Martín, 2012). In support of this hypothesis, Ferdinand *et al.* (2012) found, by using an adapted time-estimation task, that the FRN was larger to unexpected feedback, even when this feedback was of positive valence. Our findings are consistent with these results and provide significant support for the interpretation of the FRN as the manifestation of a prediction error, which indicates that an unexpected outcome, of either positive or negative valence (but see, Holroyd and Coles, 2002), has occurred, as has been formalized in the prediction of response outcome model (Alexander and Brown, 2010).

From a broader perspective, it could be argued that social rejection imposes a potential threat to the organism’s feelings of security (Panksepp, 2003), activating a set of regulatory mechanisms that, collectively, have been labelled the ‘security motivation system’ (Woody and Szechtman, 2011). This system encompasses a complex machinery of neural structures involved in threat detection and the engagement of security motivation, which then result in activating programs aimed at protecting safety. At the heart of the threat detection system is the ACC (Fiddick, 2011), which has shown to be involved in the generation of the FRN (van Veen and Carter, 2002; Ridderinkhof *et al.*, 2004) and parasympathetic cardiac control (Porges, 2001; Critchley *et al.*, 2003; Lane *et al.*, 2004; Matthews *et al.*, 2004). Specifically, the threat detection system might be tuned to environmental input that is incongruent with expectancies and, thus, potentially threatening. The FRN might be a neural manifestation of the initial detection of this incongruence, which is assumed to subsequently potentiate perceptual responsiveness making the potential threat cue more salient (Markovic *et al.*, 2014). The prolonged cardiac slowing to unexpected rejection feedback may reflect this perceptual responsiveness, in terms of an orienting response (Somsen *et al.*, 2000; Bradley, 2009) toward incongruent stimuli that are evaluated to threaten the organism’s feeling of security. Finally, the P3 response may reflect a further elaboration of the saliency of social stimuli, in that it might be a manifestation of the activity of the locus coeruleus (Nieuwenhuis *et al.*, 2005), a neural structure that has been assigned an arousal-enhancing role within the security motivation system (Woody and Szechtman, 2011). These interpretations are tentative and certainly preliminary, as they are based on a single study in young adult women only. Although some studies have shown women to be more sensitive to social rejection than men (Guyer *et al.*, 2009; Benenson *et al.*, 2013), the only study that investigated gender differences employing the social-judgment paradigm, revealed that, with respect to cardiac slowing, these differences tend to disappear after puberty (Gunther Moor *et al.*, 2014). Future studies are needed to further unravel possible gender differences in the electrocortical responses to social rejection and their implications for the here proposed social security motivation system. However, the notion of such a system may still already provide a useful conceptual framework to guide these future studies on the psychophysiology of potential threats to social belonging.

## Supplementary Data

Supplementary data are available at *SCAN* online.

## Conflict of Interest

None declared.

## Supplementary Material

Supplementary Data

## Footnotes

^1^ As women have been shown to be more sensitive to social rejection than men (Guyer *et al.*, 2009; Benenson *et al.*, 2013; Gunther Moor *et al.*, 2014), only female participants were included, to reduce interindividual variability.

^2^ Late P3 amplitudes seemed slightly right lateralized for both tasks. However, analyses over these electrode sites yielded a similar pattern of findings as compared with analyses over midline electrode sites.

^3^ The test across tasks additionally yielded a main effect of Congruency, *F*(1, 20) = 5.83, *P* = 0.025, ${\text{η}}_{p}^{2}$> = 0.23, and a Task by Feedback Type interaction effect, *F*(1, 20) = 5.62, *P* = 0.028, ${\text{η}}_{p}^{2}$> = 0.22. When collapsed over tasks, early P3 amplitudes were more pronounced following unexpected (16.71 [1.21]) relative to expected feedback (15.79 [1.06]). Effects of Feedback Type seemed specific to the age-judgment task, for which a main effect of Feedback Type was found, that was included in a Congruency by Feedback Type interaction effect (see main text).
