# Supplementary material for: Cardiac and electro-cortical concomitants of social feedback processing in women
Source: Soc Cogn Affect Neurosci. 2015 Apr 13;10(11):1506–14. doi: 10.1093/scan/nsv039 (PMC4631146; doi:10.1093/scan/nsv039)
Supplement: Supplementary Data [file supp_10_11_1506__index.html]

Cardiac and electro-cortical concomitants of social feedback processing in women — Cardiac and electro-cortical concomitants of social feedback processing in women — Supplementary Data 

# Cardiac and electro-cortical concomitants of social feedback processing in women

## Supplementary Data

files

**Files in this Data Supplement:**

- Supplementary Data - zip file
