# Supplementary material for: Cardiac and electro-cortical concomitants of social feedback processing in women
Source: Soc Cogn Affect Neurosci. 2015 Apr 13;10(11):1506–14. doi: 10.1093/scan/nsv039 (PMC4631146; doi:10.1093/scan/nsv039)
Supplement: Supplementary Data [file supp_nsv039_ns039_Supplementary_Data_Final.zip › ns039 Supplementary Data Final/nsv039_Suppl Figures.pdf]

Supplementary Figures

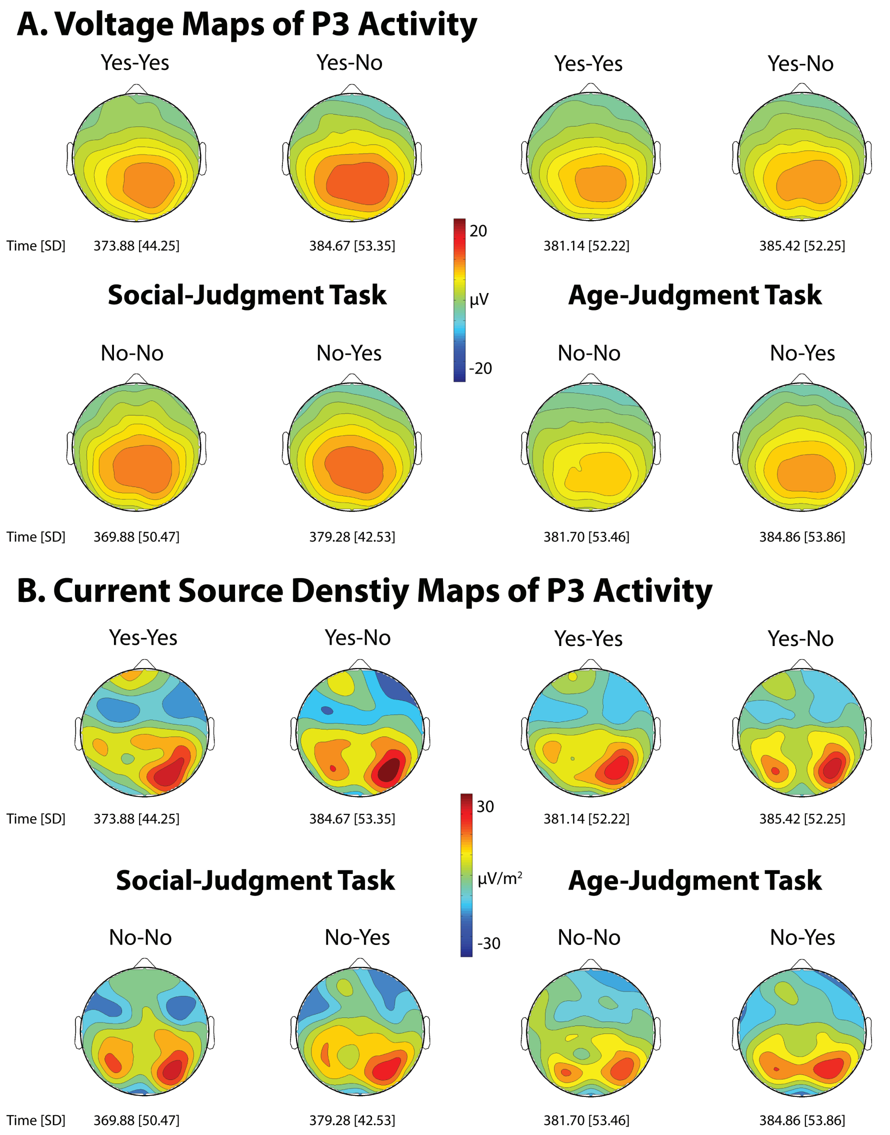

Figure S1. P3 voltage and CSD scalp maps

A: Voltage maps for P3 activity for each condition in the social- (left) and age-judgment task (right). P3 activity is plotted at P3 peak latency. B: Current Source Density (CSD) scalp maps for P3 activity for each condition in the social- (left) and age-judgment task (right). P3 activity is plotted at P3 peak latency.

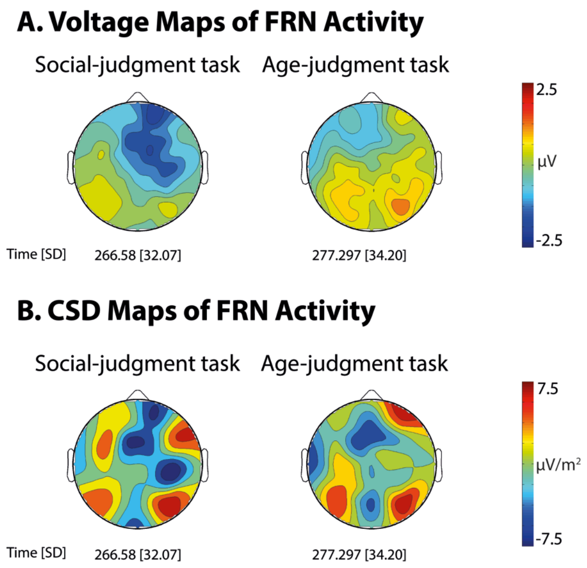

Figure S2. Voltage and CSD scalp maps of FRN activity

A: Voltage maps for FRN activity for the incongruent ('Yes'-'No', 'No'-'Yes') minus congruent ('Yes'-'Yes', 'No'-'No') contrast, plotted at grand average FRN latency, in the social- (left) and age-judgment task (right). FRN activity is defined as differential voltage at P2 and FRN latency (i.e. voltage at FRN latency minus voltage at P2 latency), for each condition, where after the incongruent minus congruent contrast was calculated. B: Current Source Density (CSD) scalp maps for FRN activity for the incongruent ('Yes'-'No', 'No'-'Yes') minus congruent ('Yes'-'Yes', 'No'-'No') contrast, plotted at grand average FRN latency, in the social- (left) and age-judgment task (right). FRN activity is defined as differential CSD at P2 and FRN latency (i.e. CSD at FRN latency minus CSD at P2 latency), for each condition, where after the incongruent minus congruent contrast was calculated.

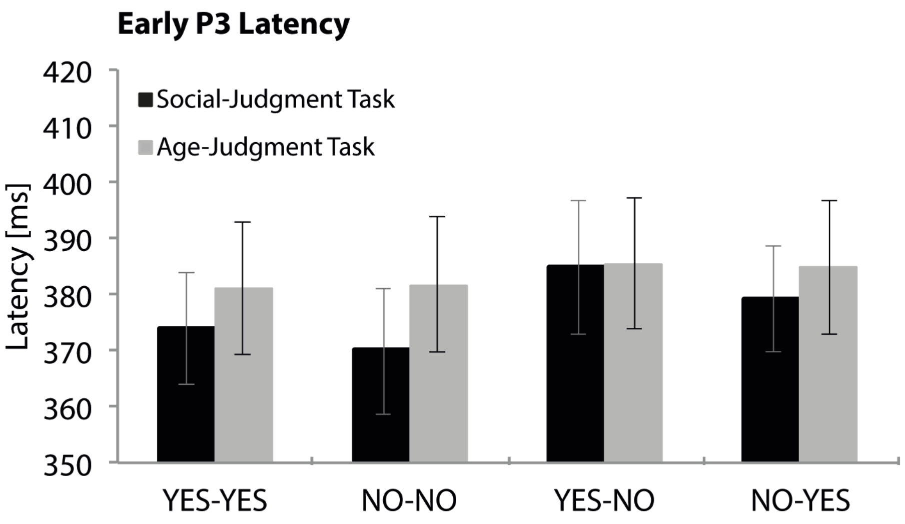

Figure S3. Early P3 latencies at Pz

Average early P3 latencies at Pz for all conditions in the social- and age-judgment task. Error bars indicate SEM.

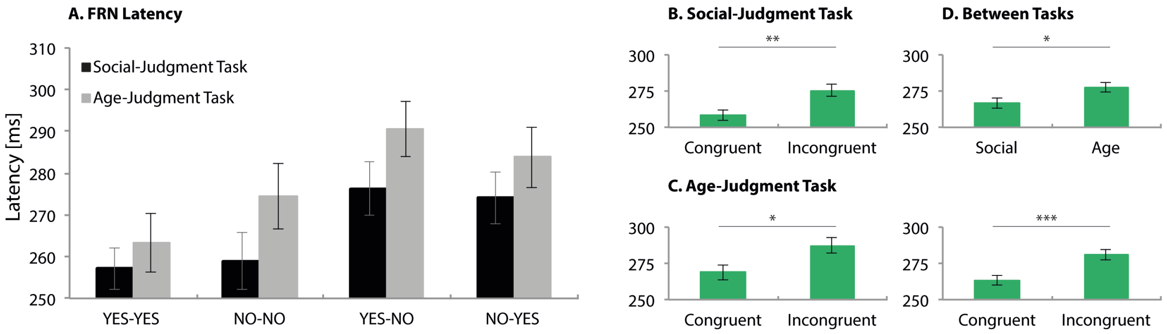

Figure S4. Feedback-related Negativity (FRN) latencies at Fz

A: Average Feedback-related Negativity (FRN) latencies at Fz for all conditions in the social- and age-judgment task. B-D: Significant main effects (for details, see Supplementary Material). * = .05 > p > .005; ** = .005 > p > .001; *** = p < .001. Error bars indicate SEM.
