# Supplementary material for: Cardiac and electro-cortical concomitants of social feedback processing in women
Source: Soc Cogn Affect Neurosci. 2015 Apr 13;10(11):1506–14. doi: 10.1093/scan/nsv039 (PMC4631146; doi:10.1093/scan/nsv039)
Supplement: Supplementary Data [file supp_nsv039_ns039_Supplementary_Data_Final.zip › ns039 Supplementary Data Final/nsv039_Suppl.pdf]

Supplementary Material

Behavioral assessment

To rule out that individual differences in cardiac and/or electro-cortical responses of interest were related to individual differences in general intelligence, participants completed the Raven's Standard Progressive Matrices (Raven, 1941). Three questionnaires were administered to investigate whether individual differences in cardiac and/or electro-cortical responses were related to individual differences in psychometric constructs, which are believed to be related to sensitivity to social acceptance and rejection. That is, 'sensitivity to peer influence' was assessed with the Dutch version of the Resistance to Peer Influence scale (RPI, Steinberg & Monahan, 2007; Sumter et al., 2009), 'level of self-esteem' was assessed with the Dutch version of the Rosenberg Self-Esteem Scale (RSES, Robins et al., 2001), and 'trait anxiety' was assessed with the Dutch version of the trait part of the State-Trait Anxiety Inventory (STAI, Spielberger et al., 1983).

Response latency

For neither the social- or age-judgment task, the paired-samples *t*-test on response latencies of 'Yes'- versus 'No'-judgments was significant, *p*s > .05.

--- Insert Figure S1 and S2 about here ---

P3 latency

Latencies of early P3 peak amplitudes at Pz are presented in Figure S3. For both the social- and age-judgment task, the ANOVA yielded no significant main- or interaction-effects, all *p*s > .05.

--- Insert Figure S3 about here ---

FRN latency

FRN latencies at Fz are presented in Figure S4. ANOVAs for both the social- and age-judgment task, only yielded a main-effect of Congruency, $F(1, 20) = 12.17$, $p = .002$, $\eta_p^2 = .38$; $F(1, 20) = 5.65$, $p = .03$, $\eta_p^2 = .22$, respectively; other *p*s > .05. For both tasks, FRN latencies were longer to unexpected (social, 275.16 [4.64]; age, 287,11 [5.32]) as compared with expected feedback (social, 258.00 [3.52]; age, 268.83 [5.19]). Subsequent test across tasks, revealed main-effects of Task, $F(1, 20) = 9.40$, $p = .01$, $\eta_p^2 = .32$, and Congruency, $F(1, 20) = 17.95$, $p < .001$, $\eta_p^2 = .47$, while the effect of Congruency did not differ between tasks, *p*s > .05. That is, FRN latency was longer in the age-judgment (277.97 [3.58]) as compared with the social-judgment task (266.58 [3.30]) and, for both tasks, following unexpected (281.13 [3.79]) relative to expected feedback (263.42 [3.35]).

--- Insert Figure S4 about here ---

Correlational analyses

For the correlational analyses an alpha of .001 was used to correct for multiple comparisons. The analyses failed to show any systematic relationship between (1) cardiac (i.e. IBI 3 response) and electro-cortical responses to social or non-social feedback, (2)

cardiac and/or electro-cortical responses to social and non-social feedback and the number of 'Yes' responses relative to the number of 'No' responses, (3) cardiac and/or electro-cortical responses to social and non-social feedback and IQ, and (4) cardiac and/or electro-cortical responses to social and non-social feedback and either resistance to peer influence or self-esteem, *p*s > .001. Lower levels of trait-anxiety (i.e. lower STAI scores), however, were associated with larger FRN amplitudes at Fz to expected social acceptance feedback (i.e. 'Yes'-'Yes' condition of the social-judgment task), $r = .673$, $p = .001$; other *p*s > .001.

Literature

Spielberger, C. D., Gorsuch, R. L., Lushene, P. R., Vagg, P. R., & Jacobs, G. A (1983). *Manual for the State-Trait Anxiety Inventory* (2nd Ed.). Palo Alto, CA: Consulting Psychologists Press, Inc.

Steinberg, L., & Monahan, K. C. (2007). Age differences in resistance to peer influence. *Developmental Psychology, 43*(6), 1531-1543. doi: 10.1037/0012-1649.43.6.1531

Sumter, S. R., Bokhorst, C. L., Steinberg, L., & Westenberg, P. M. (2009). The developmental pattern of resistance to peer influence in adolescence: Will teenagers ever be able to resist? *Journal of Adolescence, 32*(4), 1009-1021. doi: 10.1016/j.adolescence.2008.08.010

Robins, R. W., Hendin, H. M., & Trzesniewski (2001). Measuring global self-esteem: Construct validation of a single item measure and the Rosenberg Self-Esteem Scale. *Personality and Social Psychology Bulletin, 27*(2), 151-161. doi: 10.1177/0146167201272002

Raven, J. C. (1941). Standardization of progressive matrices, 1938. *British Journal of Medical Psychology, 19*(1), 137-150. doi: 10.1111/j.2044-8341.1941.tb00316.x
